# Hypothesis and Theory: Circulating Alzheimer's-Related Biomarkers in Type 2 Diabetes. Insight From the Goto-Kakizaki Rat

**DOI:** 10.3389/fneur.2019.00649

**Published:** 2019-06-26

**Authors:** Jamileh Movassat, Etienne Delangre, Junjun Liu, YuChen Gu, Nathalie Janel

**Affiliations:** ^1^Univ Paris Diderot-Sorbonne Paris Cité, Laboratoire de Biologie et Pathologie du Pancréas Endocrine, Unité de Biologie Fonctionnelle et Adaptative (BFA), UMR 8251 CNRS, Paris, France; ^2^Univ Paris Diderot-Sorbonne Paris Cité, Laboratoire Processus Dégénératifs, Stress et Vieillissement, Unité de Biologie Fonctionnelle et Adaptative (BFA), UMR 8251 CNRS, Paris, France

**Keywords:** diabetes, Alzheimer's disease, GK rat, BDNF, Dyrk1A, plasma biomarkers, tau

## Abstract

Epidemiological data suggest an increased risk of developing Alzheimer's disease (AD) in individuals with type 2 diabetes (T2D). AD is anatomically associated with an early progressive accumulation of Aβ leading to a gradual Tau hyperphosphorylation, which constitute the main characteristics of damaged brain in AD. Apart from these processes, mounting evidence suggests that specific features of diabetes, namely impaired glucose metabolism and insulin signaling in the brain, play a key role in AD. Moreover, several studies report a potential role of Aβ and Tau in peripheral tissues such as pancreatic β cells. Thus, it appears that several biological pathways associated with diabetes overlap with AD. The link between peripheral insulin resistance and brain insulin resistance with concomitant cognitive impairment may also potentially be mediated by a liver/pancreatic/brain axis, through the excessive trafficking of neurotoxic molecules across the blood-brain barrier. Insulin resistance incites inflammation and pro-inflammatory cytokine activation modulates the homocysteine cycle in T2D patients. Elevated plasma homocysteine level is a risk factor for AD pathology and is also closely associated with metabolic syndrome. We previously demonstrated a strong association between homocysteine metabolism and insulin via cystathionine beta synthase (CBS) activity, the enzyme implicated in the first step of the trans-sulfuration pathway, in Goto-Kakizaki (GK) rats, a spontaneous model of T2D, with close similarities with human T2D. CBS activity is also correlated with DYRK1A, a serine/threonine kinase regulating brain-derived neurotrophic factor (BDNF) levels, and Tau phosphorylation, which are implicated in a wide range of disease such as T2D and AD. We hypothesized that DYRK1A, BDNF, and Tau, could be among molecular factors linking T2D to AD. In this focused review, we briefly examine the main mechanisms linking AD to T2D and provide the first evidence that certain circulating AD biomarkers are found in diabetic GK rats. We propose that the spontaneous model of T2D in GK rat could be a suitable model to investigate molecular mechanisms linking T2D to AD.

## Introduction

Type 2 diabetes (T2D) and Alzheimer disease (AD) are both age-related, degenerative diseases, with increasing prevalence. Epidemiological data show strong association between AD and T2D (1). Although AD patients are not routinely evaluated for T2D or hyperinsulinemia (2), it is estimated that T2D nearly doubles the risk of dementia (3), cognitive dysfunction and AD (4). Despite the large body of epidemiological evidence linking AD to T2D, the precise molecular mechanisms underlying this association are yet unknown. Clinically, AD is a progressive neurodegenerative disease that begins with a subtle decline in the ability to encode new memories, and follows by more profound cognitive and behavioral/personality deterioration (5, 6). AD is anatomically associated with an early progressive accumulation of β-amyloid peptides (Aβ), leading to a gradual Tau hyperphosphorylation, which constitute the main characteristics of damaged brain in AD (7, 8). Apart from these processes, mounting evidence suggests that specific features of diabetes, namely impaired glucose metabolism and insulin signaling, play a key role in the brain during AD. This concept is supported by human postmortem studies showing that brain insulin resistance is consistently present in AD brains and worsens with disease progression (1, 9). The discovery of brain-specific insulin signaling deficiencies in the early stages of AD pathogenesis has led to the designation of AD as “type 3 diabetes” (10). Thus, AD lies on an intricate crosstalk between age-related metabolic, vascular, and hormonal changes that goes beyond its traditional central nervous system boundaries (6, 11).

## The Connection Between T2D and AD

There are several hypotheses in support of mechanistic links between AD and T2D. Numerous reviews have detailed the main and consolidated mechanisms linking these two conditions [review in (12–16)]. Some of the most documented mechanisms include defective insulin signaling and inflammation.

**Figure 1:**
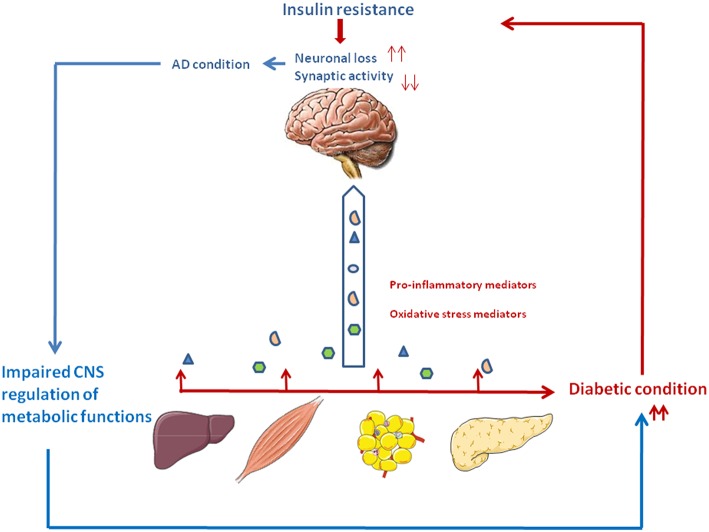
The vicious circle between type 2 diabetes and Alzheimer's disease. Type 2 diabetes is associated with relative insulin deficiency and insulin resistance. Peripheral tissues generate proinflammatory and oxidative stress mediators which can reach the central nervous system. Impaired insulin action and the excess of neurotoxic agents may lead to neuronal loss and impaired synaptic plasticity, thus participating to the cognitive and memory loss characteristics of AD. In turn, deterioration of brain areas important for glucose and energy metabolism such as the hypothalamus and hippocampus may contribute to peripheral metabolic dysfunction, thus aggravating the diabetic condition.

Importantly, T2D and AD might have a bi-directional relationship, showing both causative and consequential implications in their mutual development (Figure 1). Indeed, studies have shown that AD patients have an increased risk of developing T2D (17, 18). Studies in animal models of AD revealed increased susceptibility to develop metabolic disorders (19, 20). Several biological pathways associated with diabetes overlap with AD (21, 22). Similar to AD, pathological changes in insulin production and action occur years before patients receive a diagnosis of T2D (5, 6, 23). T2D is characterized by the association of peripheral insulin resistance and pancreatic β cell failure (24). The main organs involved in T2D development include the endocrine pancreas, liver, skeletal muscle, and adipose tissue, but also brain and small intestine. Emerging data suggest that insulin resistance (diabetic milieu) can either contribute to or serve as co-factor in its pathogenesis (1, 10). In the brain, insulin regulates peripheral Aβ and tau metabolism which influences the Aβ release in the brain by regulating amyloid precursor protein (APP) metabolism to modulate the balance between Aβ anabolism and catabolism (10). Lack of insulin or its action may link T2D to AD by modification of Aβ production and degradation.

Beyond its role in glucose metabolism in the body, insulin plays an important neurotrophic role in the nervous system [review in (12, 13)]. Numerous studies have demonstrated the implication of insulin and its signaling in neuronal survival and synaptic function and plasticity (25, 26). *In vitro*, insulin has been shown to promote neurite outgrowth in a population of dorsal root ganglions neurons (27).

Areas of the central nervous system such as the hippocampus, which are important for memory, have high expression of insulin receptor (28, 29). Therefore, impaired insulin levels or signaling in the brain can lead to neuronal and synaptic loss, and thus contribute to the development of AD and other neurodegenerative diseases.

Insulin can also have indirect effects, by inducing the expression of other neurotrophic factors. It has been shown that insulin treatment increases the expression of the brain-derived neurotrophic factor (BDNF) and tropomyosin receptor kinase B (TrkB) receptors in the hippocampus of young rats (30).

Some of the effects of insulin might also be mediated through its binding to insulin-like growth factor (IGF) receptors. IGF1 is a growth factor exerting trophic effects on neuronal regeneration. It stimulates protein synthesis in neurons, glia, oligodendrocytes, and Schwann cells, and favor neuronal survival while inhibiting apoptosis (31). Finally, insulin can promote neuronal survival by protecting the brain against neuroinflammation (32).

Inflammation plays a critical role in the pathogenesis of AD and T2D. AD pathology could be influenced by tissues involved in T2D (Liver, adipose tissue, pancreas), through the excessive trafficking of neurotoxic molecules such as pro-inflammatory mediators, generated by the diabetic condition, across the blood-brain barrier. Thus, periphery-derived pro-inflammatory molecules could aggravate AD pathogenesis in the central nervous system (Figure 1) (15, 16, 33).

However, despite intense research efforts, our knowledge of the cellular and molecular pathways linking AD and metabolic disorders remains incomplete. The understanding of the common molecular mechanisms associated with both AD and diabetes, are crucial because it could ultimately lead to the identification of common therapeutic targets for these two interconnected conditions.

## Shared Molecular Pathways Linking T2D to AD

Abnormalities in insulin/IGF signaling pathways have been shown in brains with AD (34, 35). These abnormalities were associated with reduced levels of insulin receptor substrate (IRS) mRNA, tau mRNA, IRS-associated phosphotidylinositol 3-kinase, and phospho-Akt. They also increased glycogen synthase kinase-3β (GSK3β) activity, an enzyme involved in Tau phosphorylation, and APP mRNA expression (36). Hence, the disruption of insulin functions in diabetic condition interrupts insulin signaling involved in the clearance of Aβ plaques and in neurofibrillary tangles (NFTs) pathology. This participates to the accelerated formation of neurotoxic Aβ and NFTs via various mechanisms including GSK3β and the dual-specificity tyrosine-(Y)-phosphorylation regulated kinase DYRK1A (37). Thus, insulin resistance and T2D can interact with key pathways involved in AD pathology.

Hyperphosphorylated Tau protein is the main constituent of NFTs, which alongside amyloid β plaques, have long been considered as key histopathological hallmarks of AD. Interestingly, amyloid deposition (amylin) and abnormal Tau processing may provide yet another link between diabetes and AD. Indeed, studies report a potential role of Aβ and Tau in peripheral tissues. In humans, pancreatic amyloid deposition, similar to its damaging effect in brain during AD, is associated with β cell loss and global dysmetabolism (38). High levels of phosphorylated Tau were found in pancreas from T2D patients (39). GSK3β is involved in the formation of both Aβ deposits and NFTs. GSK3β induces Tau hyperphoshorylation to form NFTs through PI3K/Akt/GSK3β signaling pathway (40). Importantly, GSK3β is also involved in many aspects of T2D pathogenesis. As a negative effector of the insulin signaling pathway, GSK3β is involved in insulin resistance (41). GSK3β expression and activity were shown to be increased in muscle of diabetic patients and was implicated in muscle insulin resistance (42). Furthermore, we and others have reported that beyond its implication in insulin resistance in target tissues, GSK3β acts as a negative regulator of β-cell growth and function, thus further implicating this enzyme in the relative insulin deficiency associated with T2D (43–46).

Peripheral insulin resistance, a hallmark of T2D, can cause brain insulin resistance via a liver/pancreas/brain axis. Elevated plasma homocysteine (Hcy) level is a risk factor for AD pathology and is also closely associated with metabolic syndrome (47–49). Elevated Hcy levels have been linked with gray and white matter volume reduction among individuals with mild cognitive impairment and AD (50). Plasma Hcy is highly dependent on intracellular Hcy metabolism in the liver and kidney, but it may also reflect one-carbon metabolism in a number of other cell types, notably in pancreas and brain. Hepatic insulin resistance during T2D leads to inflammation which could in turn results in excessive Hcy production. Cytotoxic Hcy generated in liver, traffics through the circulation following injury or cell death, and can cross the blood-brain barrier and exert neurotoxic effects by impairing central insulin signaling and activating pro-inflammatory cytokines. These abnormalities establish or help propagate a cascade of neurodegeneration associated with oxidative stress, which exacerbate brain insulin resistance, cell death, and neuro-inflammation (51) (Figure 1).

Another potentially important player in AD pathogenesis is DYRK1A (52). DYRK1A interacts with APP and APP processing by direct phosphorylation of APP at Thr-668 and indirect phosphorylation of the presenilin 1 (PS1) at Thr-354, promoting the pathological Aβ pathway (53, 54). Increased expression of DYRK1A seems to promote brain β-amyloidosis by enhancing the phosphorylation and the amyloidogenic cleavage of APP, increasing the amyloidogenic levels of Aβ40 and Aβ42 (54). It also promotes neurofibrillary degeneration directly through hyperphosphorylation of tau and indirectly through phosphorylation of alternative splicing factor, therefore participating to neurodegeneration and neuronal loss appearing in AD (54–56). Moreover, we have shown that AD patients exhibited a positive correlation between plasma DYRK1A levels and CSF tau and phosphorylated-tau proteins (57).

In recent years, increasing interest has been drawn to the role of DYRK1A in β cell biology, making it another possible molecular link between AD and T2D. Several studies show that inhibition of DYRK1A alone (58, 59) or associated with the inhibition of GSK3β (60), or with SMAD and Trithorax pathways (61) induces human β cell proliferation.

However, other data in mice model of DYRK1A overexpression showed expansion of β cell mass through increased proliferation and cell size (62), suggesting a positive effect of DYRK1A on β cell growth in this model, which contrasts with the data on human β cells cited above.

DYRK1A has been demonstrated to be involved in the cycle of Hcy (63, 64), and its overexpression was linked with BDNF reduction (65). BDNF, the most widely distributed neurotrophin in the central nervous system, has a pivotal role in maturation, synaptic connection, neuronal repair, and plasticity of the central nervous system (66). Loss of BDNF in neurodegenerative disorders is a key mediator of synaptic dysfunction, neurodegeneration and subsequent cognitive decline (67). AD subjects show reduced BDNF levels in the serum and brain as compared with healthy elderly controls (68–70). Interestingly, there was a notable increase in plasma Hcy level and significant decrease in serum BDNF level in amnestic mild cognitive impairment patients that converts to AD, especially in those with the APOE ε4 allele (71).

Higher expression of BDNF slows down cognitive decline in the elderly, especially in the setting of advancing AD neuropathology, indicating that the brain BDNF level could be used as a novel marker for evaluating AD progression (68, 72). Combined assessment of DYRK1A and the related markers BDNF and Hcy has been validated by our team, by logistic regression analysis as diagnostic marker for AD in two unrelated AD patient cohorts with age-matched controls (73).

Interestingly, several reports also documented an association between plasma BDNF and systemic or peripheral inflammatory conditions, notably T2D (74). Plasma BDNF levels were found to be decreased in T2D patients (75–78). Interestingly, the relationship between T2D, BDNF and dementia was reported in one study which demonstrated lower plasma BDNF levels in patient group with both T2D and dementia than in non-diabetic patients with dementia (79).

Treatments to alleviate brain insulin resistance, such as intranasal insulin administration, have been evaluated in mice models and AD patients (80–84). Insulinotropic hormones such as glucagon-like peptide-1 (GLP-1), have also been proposed as a treatment for neurodegenerative disorders. Indeed, exenatide, a glucagon-like peptide-1 (GLP-1) agonist used for the treatment of T2D led to improvements in motor assessments in patients with Parkinson's disease (85). The potential relevance of this drug for other neurodegenerative disorders (e.g., AD) has being assessed in pre-clinical studies. Exenatide was tested in different mice models of AD, in 3xTg-AD mice on a high-fat diet, in APP/PS1 mice, and in adult wild-type mice as a model of mid-life brain aging. Results demonstrate a beneficial effect of drug treatment not only on cognition but also on BDNF neurotrophic axis (86–88).

## Animal Models of Combined AD and T2D

Several animal models, mostly in rodents, have been designed to study the interconnection between AD and T2D. These models include high fat died-induced insulin resistance, streptozotocin-induced diabetes or monosodium glutamate (MSG)-treated rodents (89, 90). A variety of cognitive/behavioral impairments and/or histopathological defects have been reported in these studies, thus providing the experimental basis for the epidemiological data that link T2D to AD. However, most of available models have back draws since they do not replicate the progressive characteristics of T2D with a silent phase followed by the development of insulin resistance and relative insulin deficiency.

The Goto–Kakizaki (GK) rats is a spontaneous model of T2D with close similarities with human T2D (91). The chronology of the infra-clinical and clinical phases in the GK rat, ranging from primary defects in the endocrine pancreas, as early as the fetal stage, followed by a neonatal phase of pre-diabetes, and finally the occurrence of overt hyperglycemia in adult individuals has been extensively described by our team (91, 92). The relevance of the GK rat as a T2D model lies in the fact that it is a spontaneous model without genetic manipulation, in which diabetes develops through a gradual process following a well-characterized phase of pre-diabetes (93), similar to the human T2D pathology. We have previously demonstrated in GK rats, a strong association between Hcy metabolism and insulin via cystathionine beta synthase (CBS) activity, the enzyme implicated in the first step of the trans-sulfuration pathway (94). In addition to several metabolic defects, GK rats also display impairment in their learning abilities and memory capabilities. Interestingly, cognitive dysfunction in GK rats was correlated with their insulin resistance index (95). Another study have reported significant decrease in phosphorylation of Akt, as well as reduced expression of CREB, an important regulator in the expression of functional proteins associated with learning and memory in this model of T2D (96). Studies using transgenic models of AD have generated mounting evidence supporting alteration in neurogenesis (97). Previous studies by our group and other's showed that chronic hyperglycemia impairs hippocampus neurogenesis in adult diabetic GK rats (98, 99), showing similarity with defects reported transgenic mice models of AD.

Based on the literature described above, the spontaneous GK model of T2D appears as a valuable tool to investigate the relationship between T2D and AD.

## Statement of Hypothesis: Plasma Levels of DYRK1A, BDNF, and Tau Are Modified in Goto-Kakizaki Rats

Taking advantage of the characteristics of this model, we sought to analyze the circulating levels of some of the biomarkers of AD, which could potentially be related to T2D, namely DYRK1A, BDNF and Tau, in 3 and 6 months old diabetic GK rats.

**Figure 2:**
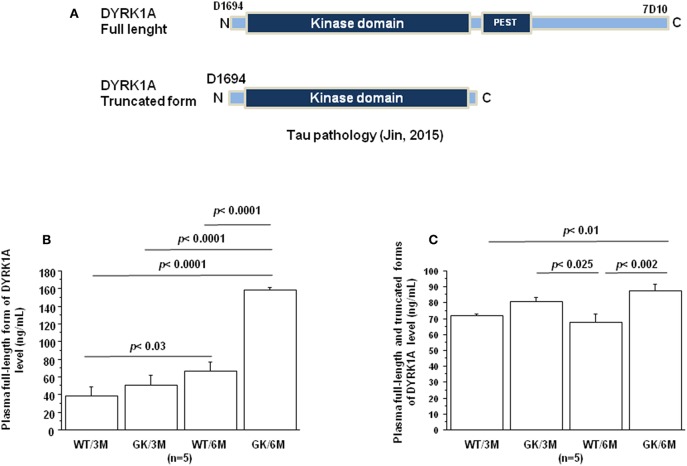
Age-dependent changes in plasma Dyrk1A levels. Blood was collected at the tail vein of Wistar and GK rats of 3 and 6 months of age, at 9:00 a.m. Analyses were performed in plasma. **(A)** Schematic representation of Dyrk1A with distinct epitopes recognized by different antibodies. Plasma levels of full-length Dyrk1A form **(B)** and full-length and truncated forms of Dyrk1A **(C)**. The DYRK1A levels were assessed by a solid phase immobilized epitope- immunoassay set up for antibody 7D10 (Abnova; immunogen: 674 aa ~763 aa) and antibody D1694 (Sigma; immunogen: 32 aa ~51 aa) (73). After removal of unbound conjugates, bound enzyme activity was assessed by use of a chromogenic substrate for measurement at 450 nm by a microplate reader (Flex Station 3, Molecular Device, San Diego, CA, USA). All the assays were performed in duplicate. For multiple pairwise comparisons between genotypes and ages, statistical analysis was done with two-way ANOVA followed by Fisher's *post-hoc* test using Statview software. The results are expressed as means ± SEM (standard error of the mean). *n* = number of rats. Data were considered significant when *p* < 0.05.

Jin et al. have reported that DYRK1A was truncated in the brains of AD patients resulting in formation of truncated forms due to increased calpain activity (100), associated with a decrease of the full-length form (Figure 2A). DYRK1A contains a PEST sequence, a signal peptide for protein degradation via calpain (101, 102). Recently, Souchet et al. found that this DYRK1A cleavage is a consequence of the amyloid pathology (103). Resulting truncated forms accumulate in astrocytes and exhibit increased affinity toward a regulator of inflammatory process (103). Here, we analyzed these different forms by the use of two different antibodies, one recognizing the full-length form, and the other the full-length and truncated forms of Dyrk1A (Figure 2A) in plasma of control Wistar (WT) and GK rats. No difference was found between WT and GK rats at 3 months (Figures 2B,C), while an increase of full-length and the truncated forms was found in GK rats at 6 months (Figure 2C). The full-length form was also increased at 6 months in WT rats compared to 3 months old WT rats (Figure 2B), suggesting an age-related effect.

**Figure 3:**
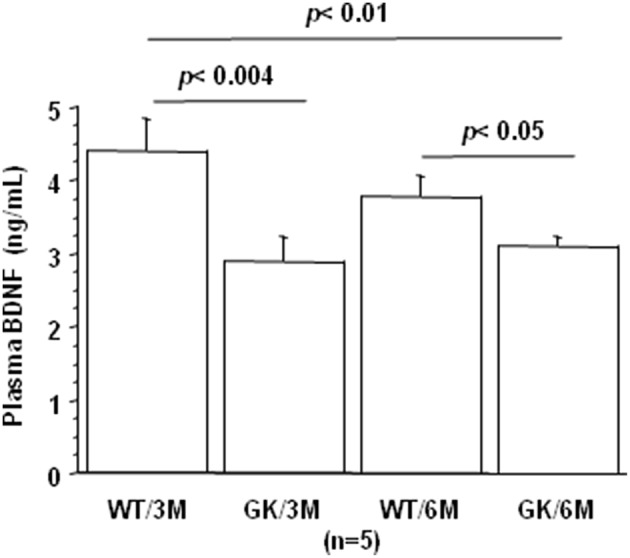
Age-dependent changes in plasma BDNF. Blood was collected at the tail vein of Wistar and GK rats of 3 and 6 months of age, at 9:00 a.m. Analyses were performed in plasma. BDNF was assessed using sandwich ELISA (ELISA E-Max, Promega, Madison, WI, USA). After removal of unbound conjugates, bound enzyme activity was assessed by use of a chromogenic substrate for measurement at 450 nm by a microplate reader (Flex Station 3, Molecular Device, San Diego, CA, USA). All the assays were performed in duplicate. For multiple pairwise comparisons between genotypes and ages, statistical analysis was done with two-way ANOVA followed by Fisher's *post-hoc* test using Statview software. The results are expressed as means ± SEM (standard error of the mean). *n* = number of rats. Data were considered significant when *p* < 0.05.

BDNF levels were decreased in plasma of GK rats at 3 and 6 months, compared to age-matched WT rats (Figure 3). This was in keeping with studies showing decreased plasma levels of BDNF in diabetic patients (75–78). There is also solid evidence demonstrating a reduction in BDNF mRNA and protein levels in AD cortex and hippocampus (104, 105), and decreased BDNF levels contribute to cognitive dysfunction in AD (66). A significant decrease in BDNF serum concentration has been found in AD patients compared with healthy controls (106). Correlations were determined by using Spearman's rank correlation, as data were not normally distributed according to Shapiro-Wilk test. A negative correlation was found between plasma BDNF and full-length and truncated forms of Dyrk1A levels (Table 1). As Dyrk1A is involved in controlling numerous pathways, this result emphasizes the role of this kinase on BDNF signaling pathways, as previously suggested by our team (65, 73).

**Table 1:** Correlations between plasma levels of Dyrk1A, BDNF, and Tau determined by Spearman's rank correlation.

	**Full-length Dyrk1A**	**Full-length and truncated forms of Dyrk1A**	**BDNF**
BDNF		*r* = −0.58	
		*p* < 0.017	
Tau		*r* = 0.758	*r* = −0.646
		*p* < 0.0007	*p* < 0.005
Tau46	*r* = 0.571	*r* = 0.646	*r* = −0.646
	*p* < 0.01	*p* < 0.002	*p* < 0.005

**Figure 4:**
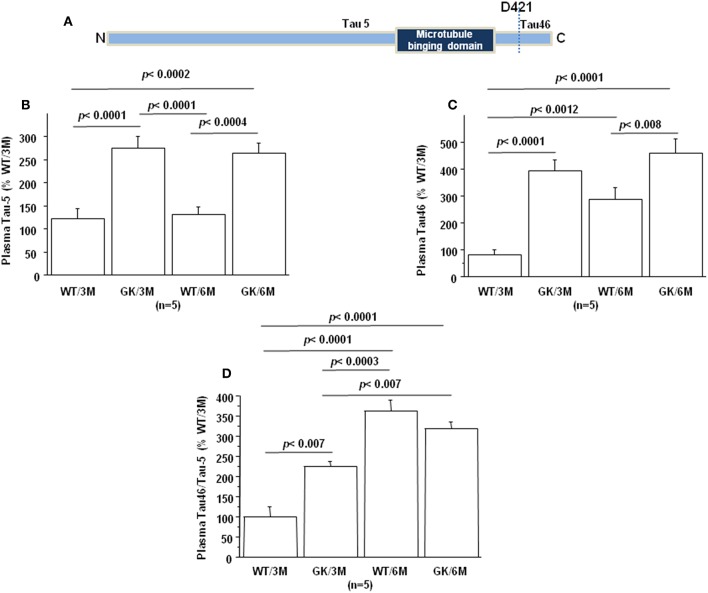
Age-dependent changes in plasma Tau. Blood was collected at the tail vein of Wistar and GK rats of 3 and 6 months of age, at 9:00 a.m. Analyses were performed in plasma. **(A)** Schematic representation of Tau with distinct epitopes recognized by different antibodies (Tau5 and Tau46) used for the truncation detection at D421. Plasma relative intensities of **(B)** Tau5 (1/1000, ThermoFisher/ AHB0042; immunogen 210 aa ~230 aa) and **(C)** Tau46 (1/1000, ThermoFisher/13-6400; immunogen 404 aa ~441 aa). **(D)** The relative intensities of Tau46 were normalized to those of Tau5. Data were normalized to the mean of wild-type rats at 3 months. For multiple pairwise comparisons between genotypes and ages, statistical analysis was done with two-way ANOVA followed by Fisher's *post-hoc* test using Statview software. The results are expressed as means ± SEM (standard error of the mean). *n* = number of rats. Data were considered significant when *p* < 0.05.

Tau protein truncated at amino acid D421 has been detected in AD (Figure 4A). This C-terminal truncation introduces a conformational change contributing to aggregation (107, 108). We therefore measured the levels of centrally-situated Tau epitope (Figure 4B) and levels of Tau 46 (Figure 4C), to evaluate the index of truncation. The index of C-terminal truncation was provided by the ratio of Tau46/Tau5 (Figure 4D). Tau levels (Tau5 immunoreactivity) increased in plasma of GK rats at 3 and 6 months, compared to age-matched WT rats. There was no difference of Tau levels between WT rats at 3 and 6 months (Figure 4A). Tau levels are correlated positively with full-length and truncated forms of Dyrk1A levels (Table 1) and negatively with BDNF levels (Table 1). Interestingly, we previously found a positive correlation between plasma Dyrk1A levels and CSF Tau proteins in AD patients (57).

An increased Tau46 immunoreactivity and Tau46/Tau 5 was found in plasma of GK rats as early as 3 months and persisted at 6 months of age compared to age-matched WT rats (Figures 4C,D). Interestingly, Tau46 immunoreactivity and Tau46/Tau5 ratio increased in an age-dependent manner within the WT group (Figures 4C,D). The Tau46 immunoreactivity also correlated positively with full-length form of Dyrk1A levels (Table 1), full-length and truncated forms of Dyrk1A levels (Table 1), and negatively with BDNF levels (Table 1). These results indicate that Tau undergoes increased C-terminal cleavage as early as 3 months in the GK rats, while this effect appears in non-diabetic Wistar rats only at 6 months.

## Conclusion

In this paper we used the type 2 diabetic GK rat as a tool to assess circulating biomarkers for AD. We show that plasma BDNF and the index of C-terminal truncation of Tau could be considered as early biomarkers, while plasma Dyrk1A could represent a late biomarker. As a spontaneous model of T2D with gradual progression, the GK rat is acknowledged as a valuable tool to study the pathogenesis of diabetes. Here we propose that the GK rat could be a new model to investigate the link between T2D and AD. It could therefore be a useful tool for pre-clinical studies to assess drug efficacy in the onset of the disease. Currently, we are addressing the question of possible abnormalities in the expression/activity of the above markers in the brain and the pancreas of the GK rat, to validate the relevance of this model as a model of T2D-associated AD. These results need to be compared with those described in validated rodent models of AD with different grade pathology. Further longitudinal studies of metabolic and cognitive parameters with pharmacological intervention are warranted to comprehend the causal relationship underlining the progression of AD and T2D.

## Data Availability

The raw data supporting the conclusions of this manuscript will be made available by the authors, without undue reservation, to any qualified researcher.

## Ethics Statement

All procedures were carried out in accordance with the ethical standards of French and European regulations (European Communities Council Directive, 86/609/EEC). Official authorization from the French Ministry of Agriculture was granted to perform research and experiments on animals (authorization number B-75-13-17) and the experimental protocol was approved by the institutional animal care and use committee of the Paris Diderot University (CEEA40).

## Author Contributions

JM and NJ designed the study, made the review of the literature, and wrote the manuscript. ED, JL, and YG performed experiments. All authors read and approved the manuscript.

### Conflict of Interest Statement

The authors declare that the research was conducted in the absence of any commercial or financial relationships that could be construed as a potential conflict of interest.
